# Antibiofilm Efficacy of Quercetin against *Vibrio parahaemolyticus* Biofilm on Food-Contact Surfaces in the Food Industry

**DOI:** 10.3390/microorganisms10101902

**Published:** 2022-09-25

**Authors:** Pantu Kumar Roy, Min Gyu Song, Eun Bi Jeon, Soo Hee Kim, Shin Young Park

**Affiliations:** Institute of Marine Industry, Department of Seafood Science and Technology, Gyeongsang National University, Tongyeong 53064, Korea

**Keywords:** *Vibrio parahaemolyticus*, quercetin, biofilm, stainless steel, hand gloves, gene expression

## Abstract

*Vibrio parahaemolyticus*, one of the most common foodborne pathogenic bacteria that forms biofilms, is a persistent source of concern for the food industry. The food production chain employs a variety of methods to control biofilms, although none are completely successful. This study aims to evaluate the effectiveness of quercetin as a food additive in reducing *V. parahaemolyticus* biofilm formation on stainless-steel coupons (SS) and hand gloves (HG) as well as testing its antimicrobial activities. With a minimum inhibitory concentration (MIC) of 220 µg/mL, the tested quercetin exhibited the lowest bactericidal action without visible growth. In contrast, during various experiments in this work, the inhibitory efficacy of quercetin at sub-MICs levels (1/2, 1/4, and 1/8 MIC) against *V. parahaemolyticus* was examined. Control group was not added with quercetin. With increasing quercetin concentration, swarming and swimming motility, biofilm formation, and expression levels of target genes linked to flagellar motility (*flaA*, *flgL*), biofilm formation (*vp0952*, *vp0962*), virulence (*VopQ*, *vp0450*), and quorum-sensing (*aphA*, *luxS*) were all dramatically suppressed. Quercetin (0–110 μg/mL) was investigated on SS and HG surfaces, the inhibitory effect were 0.10–2.17 and 0.26–2.31 log CFU/cm^2^, respectively (*p* < 0.05). Field emission scanning electron microscopy (FE-SEM) corroborated the findings because quercetin prevented the development of biofilms by severing cell-to-cell contacts and inducing cell lysis, which resulted in the loss of normal cell shape. Additionally, there was a significant difference between the treated and control groups in terms of motility (swimming and swarming). According to our research, quercetin produced from plants should be employed as an antibiofilm agent in the food sector to prevent the growth of *V. parahaemolyticus* biofilms. These results indicate that throughout the entire food production chain, bacterial targets are of interest for biofilm reduction with alternative natural food agents in the seafood industry.

## 1. Introduction

The Gram-negative pathogen *Vibrio parahaemolyticus* is frequently found in seafood [1]. During infection, it develops a biofilm, which is a collection of proteins, lipids, and polysaccharides that the microbes have self-produced and that surrounds the surface of the host [2,3]. A crucial aspect of the pathogenesis is the production of biofilm, which might increase resistance to harmful circumstances and medications. According to studies by Han et al. [4] and Almohamad et al. [5] over 60% outbreaks by *V. parahaemolyticus* biofilm by consuming contaminated seafoods. Infections with *V. parahaemolyticus* typically have self-limiting symptoms (e.g., vomiting, diarrhea, fever, nausea, chills, headaches, and watery stools) [6,7]. Although uncommon, this bacterium can cause septicemia, necrotizing fasciitis, wound infections, and even death [8,9]. The factors that play a pivotal role in the infections are adhesins (type I pilus), hemolysin, type III secretion systems (T3SS), and type VI secretion systems (T6SS) [7,10,11,12]. As a result, the aquaculture sector, the food industry, and human health could be at risk of contamination with *V. parahaemolyticus* [13].

According to the World Health Organization, O3:K6 serotypes and their variations are the most prevalent strains linked to foodborne illnesses, with *V. parahaemolyticus* being the most common cause of bacterial gastroenteritis related to the intake of seafood items globally [14]. One of the main issues for food safety and public health has been the prevalence of *V. parahaemolyticus* in the world. According to the Centers for Disease Control and Prevention (CDC) [15], *V. parahaemolyticus* causes 45,000 illnesses annually in the USA and is the most often reported in vibrio infections (Available online: https://www.cdc.gov/vibrio/faq.html (accessed on 29 August 2022) [7]. Currently, standard methods for preventing and treating *V. parahaemolyticus* contamination and infection, such as antibiotics and chemical disinfectants, are crucial [16,17]. However, studies indicate that *V. parahaemolyticus* clinical isolates and environmental isolates both shown rising antibiotic resistance globally [18,19,20]. Alternative methods of preventing bacterial contamination and illnesses are continuously being researched due to the limits of present control systems [21,22].

In comparison to their planktonic relatives, biofilms are a million times more resistant to all antimicrobial treatments [1,11]. As a result, it might be difficult to remove biofilm using regular antibiotics and cleaning products [4]. Aggressive chemicals, such as sodium hydroxide or sodium hypochlorite, are frequently employed in the food sector to reduce the negative impacts of biofilm [23,24]. However, such methods might damage the environment by corroding equipment and materials [25,26]. Therefore, it is crucial to develop a workable plan that can control and get rid of bacterial biofilm.

Biofilm is a term for bacterial growth that defends itself by routinely embedding cells in an extracellular polymeric substances (EPS), as opposed to bacterial cells that are free to move around [7,27]. This increases the bacteria’s ability to survive acquaintance to antimicrobial agents [7,28]. Multiple pathogen survival and colonization processes, including as biofilm formation and motility, are associated with pathogen infections. During the early stages of adhesion, motility is related to cell-surface attachment and the subsequent production of biofilms, and it helps bacteria withstand both the host immune system and antibacterial agents [7]. A foodborne bacterium called *V. parahaemolyticus* can grow biofilms on both biotic and abiotic surfaces, which helps it survive in conditions where food is processed [8]. Since non-motile *V. parahaemolyticus* mutants are incapable of creating biofilms, it is unclear what specific molecular foundation underlies its ability to create biofilms [9,12]. Nevertheless, flagellar motility is crucial. A number of virulence or biofilm-related genes regulate the continuous, dynamic processes that lead to the formation of biofilms, including cell attachment, EPS synthesis, resource capture, detachment, and dispersal. A variety of virulence factors, in addition to adhesion, are involved in the pathogenesis of *V. parahaemolyticus*; the expression of these factors regulates the pathogen’s virulence [7]. Several biofilm-associated genes (*vp0950*, *vp0952*, and *vp0962*) have been linked to the downregulation of virulence in *V. parahaemolyticus* biofilms [7,10]. T3SS1 and T3SS2 are expressed by numerous significant virulence-associated genes in *V. parahaemolyticus* [7]. The T3SS1 translocation effector proteins include *VOPQ* and *VPA0450*. Both clinical and environmental strains of *V. parahaemolyticus* frequently express T3SS1, which aids in the direct secretion and translocation of effector proteins into eukaryotic cells [7,10]. The inositol polyphosphate 5-phosphatase *VPA0450* and the pore-forming effector *VOPQ* both have the ability to cause autophagy when an infection is taking place. On chromosome 2 of *V. parahaemolyticus*, the *vp0952*, *vp0950*, and *vp0962* genes encode proteins that are homologs of biofilm-associated proteins [10]. The two genes of *luxS* and *aphA* have been intensively explored, with a focus on their role in quorum-sensing regulation, a challenging cell-to-cell procedure that enables bacteria to observe their environment and cooperate [7,10]. The regulation of the development of *V. parahaemolyticus* biofilms have also been linked to the *luxS* gene [7,10]. According to studies [18,29], *V. parahaemolyticus* can form biofilms on a variety of biotic or abiotic surfaces and interfaces, including seawater and marine organisms (shrimp, fish, crab, shellfish, stainless steel, hand gloves, etc.) [7]. This contamination of the sea and seafood leads to cross-contamination during the processing or preparation of food [7,30]. Cross-contamination may be a significant source of human diseases, according to reports [1,11,31]. The development of biofilms on or in seafood may have a significant role in the spread of *V. parahaemolyticus* and the subsequent illnesses [32]. Biofilm represents an important target for the reduction in contamination and illnesses brought on by *V. parahaemolyticus*.

Microbial biofilms, where bacteria reside, provide them with protection from physical injury, desiccation, and antibiotics [33]. According to numerous studies, foodborne pathogens persist as biofilms on food-contact surfaces (e.g., plastic, steel, glass, and rubber) and have an impact on the quantity, quality, and safety of food products [34,35,36,37]. Additionally, they destroy surfaces and equipment, contaminate food on a constant basis, pose a significant risk to public health, and their control is a significant barrier in the food production chain [38]. To prevent foodborne infections, natural plant extracts and macroalgae extracts antimicrobial compounds are typically regarded as secure, efficient, and environmentally friendly [39,40,41]. Certain plant extracts have long been used widely for food preservation and disease prevention due to their wide spectrum of activity against different bacterial and fungal infections [7,40,42].

One of the preventative strategies for improving food quality and safety is the use of substances that block quorum sensing (QS) [43,44]. One of them targets QS, a mechanism that allows cells to communicate with one another and allows germs to survive under adverse conditions [37]. When bacterial concentrations approach a predetermined concentration threshold, signaling molecules or auto-inducers are secreted, which control the expression of virulence genes at bacterial densities [37,45]. Numerous virulence factors, such as the production of nuclease, hemolysin, lipase, protease, prodigiosin, as well as the development of biofilms and motility, are regulated by QS [37,45]. QS in a number of bacteria can be disrupted by phenolic chemicals generated from plants [43]. Plant compounds are an alternative control method against *V. parahaemolyticus* biofilms and one of the most investigated flavonoid molecules having functional characteristics in this context is quercetin. Flavonoids have become well known for having anti-inflammatory, antioxidant, antibacterial, and anticancer properties [45] in addition to their potential QS system inhibitory properties [46,47]. Many fruits and vegetables, including apples, tea, onions, red grapes, berries, tomatoes, and tea, contain quercetin, a flavonoid-based compound [48]. Due to its anti-inflammatory, anticancer, and neuroprotective properties, it has a wide range of applications [49,50]. Owing to its three-ring structure with five hydroxyl groups, it possesses especially strong antioxidant capabilities [37,45,49]. Antioxidants can reduce oxidative stress and prevent biofilm formation by eliminating reactive oxygen species (ROS) accumulated in bacterial cells [37,45]. As a result, antioxidants are potent antibiofilm agents [49,51]. One of the primary processes by which oxidative stress induces bacteria to develop biofilm as a survival strategy. Additionally, it has already been demonstrated that quercetin has antibacterial properties against both Gram-positive and Gram-negative bacteria [48], including *Staphylococcus aureus* [48,52], *Escherichia coli* [48,53], and *Pseudomonas aeruginosa* [48,54]. Therefore, antibiofilm activity is very crucial to make the food safety from microbial contamination. It is most likely that this plant extract affects certain biofilm formation processes, such as initial adhesion or EPS generation, because the antibiofilm action is shown at non-lethal dosages. Studies have shown that the flavonoids rutin and catechin, as well as the phenolic acids gallic, ferulic, and caffeic acids, inhibit the capacity of foodborne pathogens to cling to stainless steel, hand gloves, and silicon rubber surfaces [55]. This outcome could be explained by their capacity to inhibit bacterial migration and change the physicochemical properties of various substrates (e.g., surface charge and hydrophobicity). As a result, quercetin has an impact on how well foodborne pathogens control biofilms in the food industry. Because flavonoids have changed the pathogenicity of foodborne pathogens, we speculate that quercetin may have an effect on the growth of this pathogen’s biofilm.

However, no research has specifically inspected quercetin’s antibiofilm activity against *V. parahaemolyticus*. In the current investigation, quercetin at sub-MIC was tested for its ability to suppress *V. parahaemolyticus* biofilm formation on food-contact surfaces, as well as QS-regulated behaviors and flagella motility, as well as its impact on virulence and QS gene expression.

## 2. Materials and Methods

### 2.1. Bacterial Strain Culture and Growth Conditions

*Vibrio parahaemolyticus* was collected from American Type Culture Collection (Manassas, VA, USA) strain (ATCC 27969) and used for the biofilm-forming assays. The bacteria were cultured in tryptic soy broth (TSB, BD Difco, Franklin Lakes, NJ, USA) with 2.5% NaCl at 30 °C for 24 h followed by another sub-culture at 18 h [55]. Briefly, stock solutions of the bacteria strains (cell density: 10^8^–10^9^ CFU/mL) were stored in phosphate-buffered saline (PBS; Oxoid, Basingstoke, UK) containing 30% glycerol in a deep freezer at −80 °C. First, 100 μL of bacteria was inoculated into 10 mL of tryptic soy broth (TSB; BD Difco, Detroit, MI, USA) and cultured at 30 °C and 200 rpm in a shaking incubator (Vision Scientific, VS-8480, Seoul, Korea). After 24 h, 100 μL was taken from the culture medium and inoculated in 10 mL of fresh TSB, then placed in the shaking incubator under the same conditions as the previous day. The culture was centrifuge (11,000× *g* for 10 min) and washed two times with phosphate-buffered saline (PBS; Oxoid, Basigstoke, England). After that, peptone water (PW; Oxoid, Basingstoke, England) was added to the final bacterial solution to reach the 10^5^ log CFU/mL of bacteria. The formation of biofilms on surfaces of SS and HG was then accomplished using these inoculums (10^5^ CFU/mL).

### 2.2. Preparation for Food-Contact Surfaces

With few modifications, sample preparation was performed as explained in our earlier investigations [55]. Using a sterile scissors, hand gloves latex (HG, Komax Industrial Co., Ltd., Seoul, Korea) were cut into 2 × 2 cm^2^ coupons and stainless-steel coupons (2 × 2 × 0.1 cm, type: 304) were used. Following the removal of any dirty, the coupons were cleaned with sterile distilled water (DW). The coupons were sterilized by UV-C light for 15 min on each side [55]. The coupons were dipped into 10 mL of TSB, infected with bacteria (10^5^ CFU/mL), and then incubated for 24 h at 30 °C without shaking to test for the further experiment.

### 2.3. Quercetin Preparation and Determination of Minimum Inhibitory Concentration (MIC)

From Sigma-Aldrich, we collected quercetin (Q-4951) (St. Louis, MO, USA). After being dissolved in dimethyl sulfoxide (DMSO, Sigma-Aldrich, St. Louis, MO, USA), the product was used to make a stock solution with a concentration of 1 mg/mL. The MIC was verified and very slightly modified from previous study [37]. A two-fold serial dilution approach using TSB was used to establish the minimum inhibitory concentration (MIC) of quercetin against *V. parahaemolyticus*. A total of 100 µL of quercetin serially diluted with TSB and 100 µL of bacterial suspension (10^5^ log CFU/mL) were combined in 96-well plates (Corning Incorporated, Corning, Inc., Corning, NY, USA). Each well had a total amount of 200 µL. A microplate reader (Spectra Max 190, Sunnyvale, CA, USA) was used to measure absorbance (600 nm) while the plates were kept in a 30 °C incubator for 24 h. After an overnight incubation at 30 °C, aliquots (100 µL) taken from the wells that had no discernible growth were plated on Vibrio CHROMagar (CHROMagar, Paris, France) plates and the number of colonies counted. Triplicates of this experiment were performed.

### 2.4. Analysis of Motility

Motility experiments were carried out in this study with minor variations from those previously published [37]. This test was conducted to verify the effect of quercetin on the two forms of *V. parahaemolyticus* motility (swimming and swarming). Bacto agar (BD Dicfo, Franklin Lakes, NJ, USA) was mixed with TSB at a rate of 0.3% and 0.5% to provide the media for the swimming and swarming studies, respectively. Each plate was filled with the autoclaved medium. Quercetin was added (0, 110, 55, 27.5 µg/mL) and thoroughly mixed in before it set. For swimming and swarming, it was incubated at 30 °C for 13 and 48 h, respectively. The motility diameter (migration of bacteria via the agar) was evaluated in mm, then expressed the motility as % (calculated control as 100%).

### 2.5. Biofilm Formation and Detachment Process

With slight adjustments, the procedure was carried out as previously described [37]. The MIC in this study was 220 µg/mL, and the inhibiting effect of biofilm was seen at sub-MIC levels, which may not have killed the bacteria but affected their virulence factor. Control, 1/8, 1/4, and 1/2 MIC concentrations were used in this study. In a 50 mL conical tube with 10 mL TSB (adjusted with quercetin and bacterial suspension), quercetin, and 100 µL of bacterial suspension (10^5^ log CFU/mL), the prepared samples were placed. They were then thoroughly combined with a vortex mixer (Scientific Industries, SI-0256, Bohemia, NY, USA) before being incubated for 24 h at 30 °C. After the biofilm formation, the coupons were washed twice with distilled water (DW) [37,45]. After washed, the coupons were placed in 10 mL peptone water (PW; BD Diagnostics, Franklin Lakes, NJ, USA) 50 mL Falcon tube, which contained 10 glass beads [11,37]. This bacterial suspension sample was serially diluted before being placed into Vibrio CHROMagar plates as an inoculum. The number of colonies on the plates was counted after they had been kept in a 30 °C incubator for 24 h. After subtracting the populations of each concentration (0, 1/8, 1/4, and 1/2 MIC) from the populations of each group, we were able to calculate the inhibition values and measured as log CFU/cm^2^.

### 2.6. Confirmation of Biofilms Inhibition by Field Emission Scanning Electron Microscopy (FE-SEM)

To confirm the biofilm inhibition by quercetin (Control, 1/4, and 1/2 MIC) on food-contact surfaces (HG) were observed by FE-SEM. With minor modifications, samples were prepared according to a previous study [37]. Briefly, the samples were fixed with 2.5% glutaraldehyde in PBS and stored at room temperature for 4 h and after that treated with ethanol (50, 60, 70, 80, 90% for 15 min serially) and 100% for 15 min two times. Then the samples were dehydrated with soaking (33, 50, 66, and 100% hexamethyldisilazane in ethanol) for 15 min serially. The samples were dried in a fume-hood for 3 h and platinum sputed-coated (Q150T Plus, Quorum, UK) and observed by FE-SEM (Hitachi/Baltec, S-4700, Tokyo, Japan) [45,56].

### 2.7. RNA Extraction, cDNA Synthesis, and Real-Time PCR (RT-PCR)

With a few minor adjustments, the experiment was carried out as previously described [37]. The test was carried out to confirm quercetin’s impact on *V. parahaemolyticus* pathogenicity, motility, and QS gene expression. Each Falcon^®^ tube containing 10 mL of TSB with quercetin received an inoculation of the bacteria (10^5^ log CFU/mL). They were kept in an incubator at 30 °C for 24 h. Total RNA was collected using the RNeasy Mini kit (Qiagen, Hilden, Germany) followed by the manufacturing protocol. Using a Maxime RT PreMix (Random Primer) kit (iNtRON Biotechnology Co., Ltd., Seoul, Gyeonggi-do, Korea), cDNA was produced after the RNA yield and purity were assessed using a spectrophotometer at 260/280 nm and 260/230 nm (NanoDrop, Bio-Tek Instruments, Chicago, IL, USA) [57]. Table 1 listed the primers. The housekeeping gene was 16S rRNA. In a total volume of 20 µL, the cDNA sample was combined with the appropriate primers and Power SYBR Green PCR Master Mix (Applied Biosystems, Thermo Fisher Scientific, Warrington, UK). A CFX Real-Time PCR System (Bio-Rad, Hercules, CA, USA) was used to perform the RT-PCR analysis. Utilizing 2X Real-Time PCR Master Mix and 1 µL of cDNA as a template, RT-qPCR was carried out. A CFX Real-Time PCR System was used to conduct the real-time PCR. Initial denaturation for the PCR reaction occurred at 95, 50, and 72 °C for 20 s each [57,58,59]. After PCR cycling was complete, we collected cycle threshold (Ct) values to confirm the specificity and conducted 2^−^^△△Ct^ method analysis [60,61,62,63].

### 2.8. Statistical Analysis

At least three times each of the experiments were performed. All data were expressed as mean ± standard error of mean (SEM). Statistical significance was set at *p* < 0.05 when Ducan’s multiple-range test and one-way ANOVA were performed using SAS software version 9.2 (SAS Institute Inc., Cary, NC, USA) to determine the significance.

## 3. Results

### 3.1. MIC Determination

**Figure 1:**
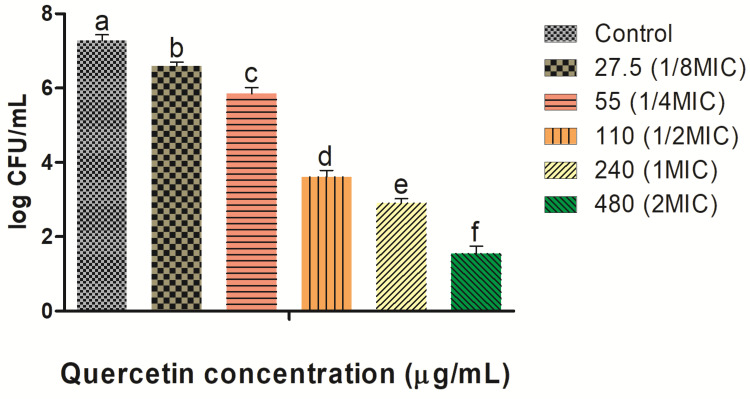
Effects of quercetin against *Vibrio parahaemolyticus* planktonic cells with different concentrations (μg/mL) were used in this study. Data are represented as mean ± SEM of three independent replicates. ^a–f^ Values with different letters differ significantly different by Duncan’s multiple-range test (*p* < 0.05).

Quercetin is dose-dependent from species to species. The MIC was established as the lowest quantity with no visible growth bacterial growth. Quercetin was evaluated for its inhibitory activity against the growth of *V. parahaemolyticus*. Therefore, further experiments determined the MICs of quercetin. With various concentrations of quercetin (from 27.5 to 480 MIC), we found that until 220 MIC, the quercetin did not significantly affect (*p* ≥ 0.05) bacterial growth. Therefore, the sub-MIC range was determined as 27.5–110 MIC, and this concentration range was used in all experiments henceforth. The outcomes indicated that the MICs of quercetin against *V. parahaemolyticus* (ATCC 27969) were 220 µg/mL (Figure 1). For further experiments in this study, different sub-MICs (1/8, 1/4, and 1/2 MICs) of quercetin were used.

### 3.2. Swimming and Swarming Motility Assays

**Figure 2:**
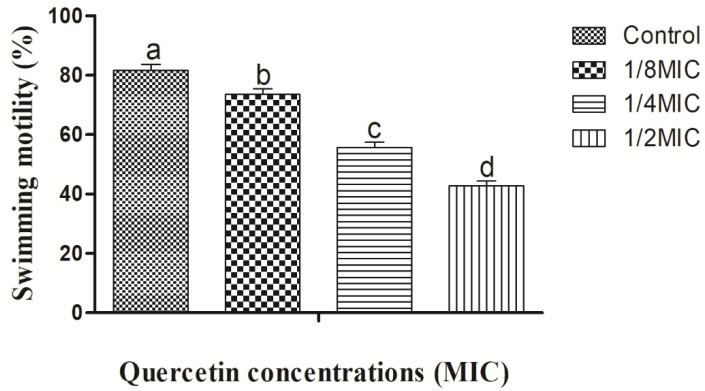
Swimming motility assay for *Vibrio parahaemolyticus* with sub-MICs of quercetin (μg/mL). Data are represented as mean ± SEM of three independent replicates. ^a–d^ Values with different letters differ significantly different by Duncan’s multiple-range test (*p* < 0.05).

**Figure 3:**
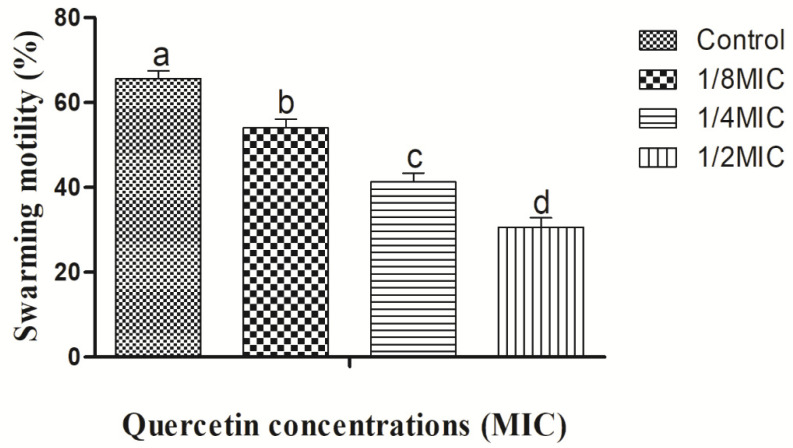
Swarming motility assay for *Vibrio parahaemolyticus* with sub-MICs of quercetin (μg/mL). Data are represented as mean ± SEM of three independent replicates. ^a–d^ Values with different letters differ significantly different by Duncan’s multiple-range test (*p* < 0.05).

For the formation of biofilms, bacterial flagella must be mobile. *Vibrio parahaemolyticus* flagella can be verified by swimming and swarming assays, in particular. The impact of quercetin on inhibiting *V. parahaemolyticus* motility is depicted in Figure 2 and Figure 3. Quercetin reduced *V. parahaemolyticus* motility by 89 and 51%, respectively, in the swimming experiment when compared to the control at 1/8 and 1/2 MIC. Figure 2 depicts the quercetin’s inhibition of *V. parahaemolyticus*.

Quercetin thereby reduced *V. parahaemolyticus* motility by 78% and 44% at 1/8 and 1/2 MIC compared to control, respectively (Figure 3). Thus, in this experiment, as quercetin concentration increased, swimming and swarming motility became more inhibited. Particularly in comparison to the control group, motility was significantly different with 1/2 MIC of quercetin.

### 3.3. Eradication Effect of Food Additive Quercetin on Food-Contact Surfaces against V. parahaemolyticus

**Figure 4:**
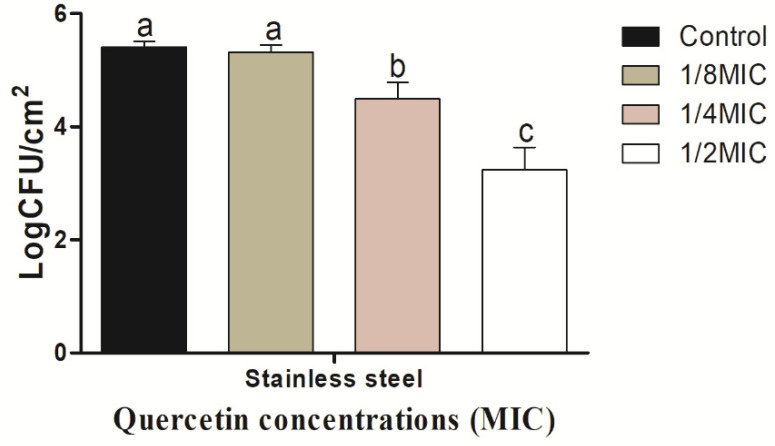
Inhibition of *Vibrio parahaemolyticus* biofilm formation (24 h) on stainless steel by sub-MICs of quercetin (μg/mL). Data are represented as mean ± SEM of three independent replicates. ^a–c^ Values with different letters differ significantly different by Duncan’s multiple-range test (*p* < 0.05).

The *V. parahaemolyticus* biofilm on the SS coupon shows in Figure 4 to be inhibited by quercetin.

**Figure 5:**
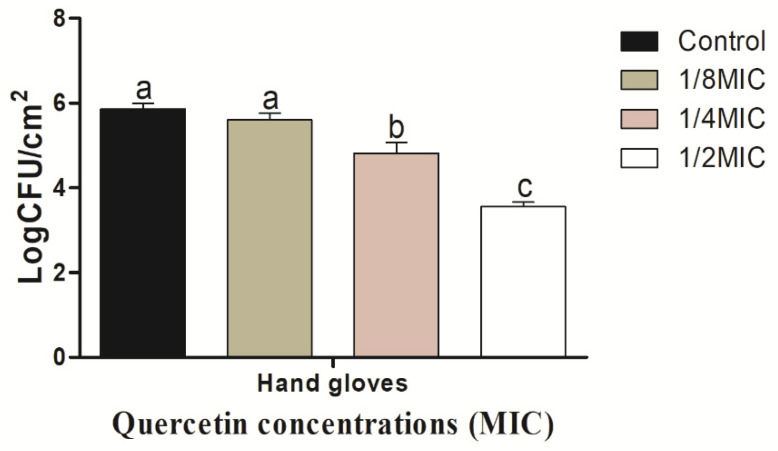
Inhibition of *Vibrio parahaemolyticus* biofilm formation (24 h) on hang gloves by sub-MICs of quercetin (μg/mL). Data are represented as mean ± SEM of three independent replicates. ^a–c^ Values with different letters differ significantly different by Duncan’s multiple-range test (*p* < 0.05).

As quercetin content increased, the biofilm-inhibiting impact grew as well. The *V. parahaemolyticus* biofilm inhibition values on the SS surfaces were 0.10, 0.92, and 2.17 log CFU/cm^2^, respectively, at quercetin quantities of 1/8, 1/4, and 1/2 MIC. Comparing these values to the control and other MIC groups, they were significantly (*p* < 0.05) suppressed at 1/2 MIC. On the HG surface, *V. parahaemolyticus* biofilm is shown in Figure 5 to be inhibited by quercetin. The *V. parahaemolyticus* biofilm inhibitory values were 0.26, 1.40, and 2.31 log CFU/cm^2^ at 1/8, 1/4, and 1/2 MIC quercetin concentrations, respectively. Compared to the control and other MIC groups, 1/2 MIC significantly inhibited biofilm formation (*p* < 0.05).

### 3.4. Biofilm Inhibition Confirmation by Quercetin under FE-SEM

**Figure 6:**
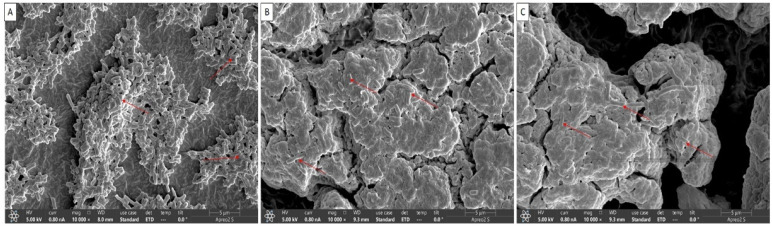
Representative scanning electron micrographs of *V. parahaemolyticus* biofilms formation in the presence of sub-MICs of quercetin on the hand glove surfaces: (**A**) Control (0% quercetin); (**B**) 1/4 MIC; (**C**) 1/2 MIC.

The visual confirmation of biofilm inhibition by quercetin is shown in Figure 6. The biofilms were architecturally structured with intact cell-to-cell contacts in control samples. Smooth and regular cells with intact cell membranes were observed in both the control (Figure 6A) and the quercetin-supplemented groups (Figure 6B,C). The rough and uneven appearance of quercetin-treated bacterial cells indicated that the cells had lost their usual shape (Figure 6B,C). Red color marked indicated attachment of biofilms cells in control (5A) and single, and lysis of biofilms in quercetin-treated samples (Figure 6B,C).

### 3.5. Motility, Virulence, Biofilm Formation, and QS Sensing Relative Gene Expression Pattern

**Figure 7:**
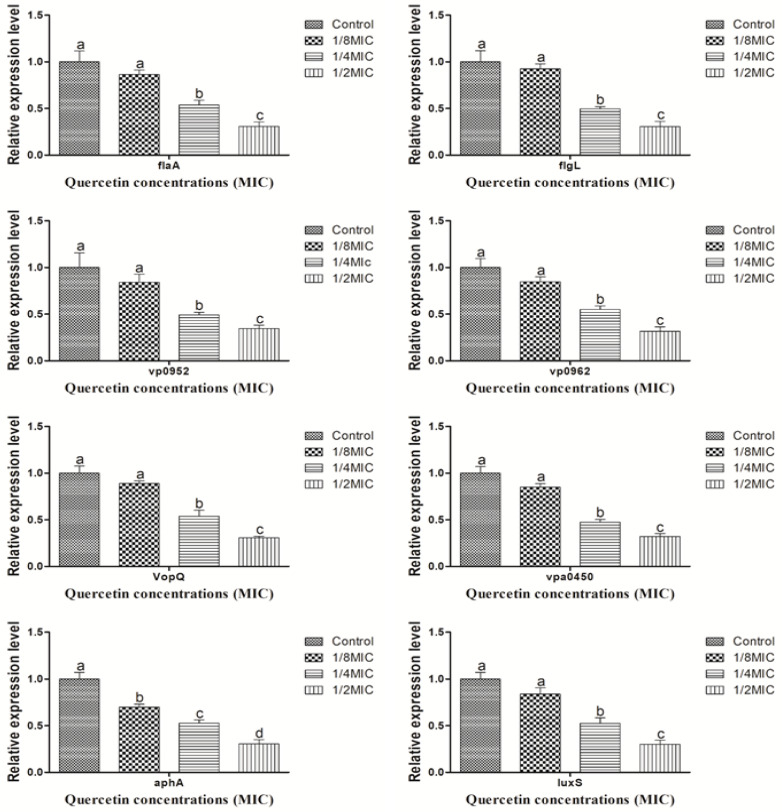
Relative expression levels of *flaA*, *flgL*, *vp0952*, *vp0962*, *VopQ*, *vpa0450*, *aphA*, and *luxS* genes in *Vibrio parahaemolyticus* supplemented with sub-MICs of quercetin. ^a–d^ Different superscript letters indicate significant differences (*p* < 0.05) with three independent replicates.

Figure 7 shows the expression of *V. parahaemolyticus* motility, virulence, biofilm formation, and QS factor, as determined by RT-PCR in the sub-MIC of quercetin (from 0 to 110 µg/mL). At the various sub-MIC concentrations of quercetin, gene expression was considerably downregulated (*p* < 0.05).

## 4. Discussion

Plant-derived natural compounds offer a potentially practical way to go beyond bacterial biofilm inhibitory mechanisms and restore quercetin potency. Because they contain quercetin, plant extracts could be considered food elements rather than food additives. Unspecific protein kinase enzyme inhibitors include quercetin. In 2010, the FDA authorized the use of high-purity quercetin at levels up to 500 mg as an ingredient in a number of specific food categories [37]. The goal of the current investigation was to determine whether quercetin at sub-MIC levels could be used to inhibit the growth of *V. parahaemolyticus*. Against *V. parahaemolyticus*, quercetin has antibacterial efficacy, which we describe in our study. We revealed that there was a dose-dependent bactericidal effect of quercetin against *V. parahaemolyticus,* as well as a considerable biofilm formation inhibition caused by quercetin using a variety of techniques, including bacterial motility and growth of biofilm. In addition to suppressing bacterial growth, quercetin also reduced *V. parahaemolyticus*-induced pathogenicity, biofilm formation, flagellar motility, and QS gene expression.

The MIC of quercetin was determined to be 80 µg/mL for *Pseudomonas aeruginosa* and *Klebsiella pneumoniae*, 120 µg/mL for *Chromobacterium violaceum*, 250 µg/mL for *Salmonella Typhimurium*, and 95 µg/mL for *Yersinia enterocolitica* [37,45,64]. By encouraging surface adhesion, swimming and swarming locomotion affect bacterial biofilm development. Our results clearly show that quercetin dramatically decreased the test pathogens’ flagella-mediated motility when compared to the control (Figure 2 and Figure 3). The outcomes are analogous to those reported by Damte et al. [65], who found that plant extracts can reduce *Pseudomonas* swarming motility by 71%. Another finding was that cinnamaldehyde prevented *E. coli* swarming by reducing biofilm development, according to Niu and Gilbert [66]. Similarly, quercetin reduced the motility at swimming (77 and 76%) and swarming (55 and 54.5%) against *S. Typhimurium* [37,45]. As a result, quercetin seems to inhibit the ability of foodborne pathogens to attach to surfaces, hence reducing the formation of biofilms. Another important aspect of pathogenicity is bacterial motility, which includes swimming and swarming. The examined bacteria’ motility was greatly reduced in this instance by quercetin.

The formation of biofilms is among the most essential elements of a foodborne bacteria’s pathogenicity. QS is one of the crucial factors in the formation of biofilms [67]. Thus, disrupting the signal-mediated QS system may control the development of biofilms. The study’s findings demonstrated that quercetin effectively decreased the biofilm development in test pathogens at all tested concentrations. Our results are in line with those previously reported [37,45], which claimed that as compared to control, quercetin (125 µg/mL)-treated foodborne pathogens *S. Typhimurium* rarely form biofilms on food and food-contact surfaces. Another researcher reported [68] 0.2 mM of quercetin was used against *Listeria monocytogenes* biofilm formation, which was necessary for the observation of changes brought on by quercetin [37]. In order to rule out any interference from quercetin (0.2 mM) on planktonic populations during the experiment, its impact on *L. monocytogenes* planktonic growth kinetics was also assessed [37]. Because planktonic cells in the bulk medium continuously deposit onto layers of attached cells throughout normal development, it is important to recognize their role in biofilm formation. The results showed that the flavonoid quercetin prevented the development of *L. monocytogenes* biofilm and suggests that quercetin affects biofilm formation mechanisms other than cell division [37,65]. However, increasing quercetin levels had an impact on the formation of biofilms, as 1.96 and 3.21 Log10 CFU/cm^2^ of viable surface-associated cells were decreased at concentrations of 0.2 and 0.4 mM, respectively, with a significant reduction (*p* < 0.05) in quercetin levels [37,68]. Additionally, at sub-MIC of quercetin, the biofilm was more inhibited by quercetin on food-contact surfaces (SS and HG) surfaces (Figure 4 and Figure 5). Vibrio can attach to plastic surfaces and create a biofilm, making the use of plastic cutting boards and cooking raw foods extremely prone to cross-contamination [37,69,70]. Additionally, compared to glass and SS surfaces, which are hydrophilic materials, plastic is more likely to allow *Salmonella* germs to stick to them [37,45,71]. Therefore, it is crucial to avoid contaminating the plastic cutting boards used while preparing or processing food because this leads to vibriosis. Other authors looked at the efficacy of quercetin in inhibiting the formation of biofilms in *Staphylococcus epidermidis* [49]. Quercetin inhibited the growth of biofilms in a concentration-dependent manner. Quercetin reduced the growth of *S. epidermidis* biofilm by 90.5 and 95.3% at 250 and 500 µg/mL concentrations, respectively [49]. Bacteria lose their normal structure as a result of quercetin’s potential disruption of cell-to-cell connections [37]. These intercellular connections encourage bacterial colonization and the formation of orderly biofilms. The cells of the biofilm become separated when these connections are disrupted and are then easily eliminated by washing [11]. According to FE-SEM images of *V. parahaemolyticus,* quercetin disrupts cell-to-cell connections (Figure 6), which is consistent with previous studies [37,45].

The pathogenicity, biofilm development, and physiological characteristics of *V. parahaemolyticus* depend on a variety of genes. To assess the effectiveness of quercetin, we examined the gene expression profiles for QS (*luxS* and *aphA*), motility (*flaA* and *flgL*), virulence (*VopQ* and *Vpa0450*), and biofilm-related (*vp0952* and *vp0962*) in *V. parahaemolyticus*. Pathogenicity, QS, and virulence elements processes are interconnected. Preventing or limiting QS production is an emerging strategy for preventing biofilm formation, reducing pathogenic infections, and ensuring food safety. When there is an accumulation of ROS inside the cell, oxidative stress results [72]. By enhancing microbial population adaptation and survival protection, oxidative stress contributes significantly to the production of biofilms [51]. Not just in human cells but also in microbes, ROS are crucial signaling molecules [45]. To keep a healthy redox cycle going and to encourage microbial adhesion, ROS can act as both intracellular and extracellular stimulants [37,51]. This will eventually result in the formation of biofilms. There may be an accumulation as a result of a disruption in the redox cycle [37]. By generating ROS within cells and weakening the membrane integrity of bacterial cells, the antioxidant quercetin prevents the formation of biofilms [50]. Quercetin significantly reduced both forms of motility as well as the transcription of the *flaA* and *flgL* genes in the current investigation (Figure 7). These genes are connected to the control of flagella synthesis and structure in *V. parahaemolyticus* [8]. For instance, the *flaA* gene, which encodes polar flagellin, contributes to swimming motility, and the lateral flagellar gene system of *V. parahaemolyticus*, which allows bacteria to spread out and colonize surfaces (swarming), contains the *flgM* gene, which encodes anti-28 [8,73]. These results were in line with those of an earlier study [74], which found that thymoquinone decreased the expression of genes related to flagella production and hindered the motility of *V. parahaemolyticus*. A number of virulence factors, in addition to adhesion, are involved in the pathogenesis of *V. parahaemolyticus*, and their expressions affect the pathogen’s pathogenicity. Specifically for the *VopQ*, *vpa0450*, *vp0952,* and *vp0962* genes, our findings showed that quercetin dramatically reduced the expression of a number of virulence and biofilms-related genes (Figure 6). On chromosome 2 of *V. parahaemolyticus*, the genes *vp0950*, *vp0950*, and *vp0962* all encode proteins that are similar to those found in biofilms [7,75]. The transcription of the genes *ompW, luxS*, and *aphA*, which had previously been downregulated by citral in a previous study, was likewise drastically reduced by natural plant extracts [7,76]. The two genes *luxS* and *aphA*, which have both been extensively studied, play a major role in the control of quorum-sensing, a difficult cell-to-cell procedure that allows bacteria to monitor their environment and collaborate [7,77]. The *luxS* gene has also been shown to control the production of thermostable direct hemolysin (TDH) and the growth of *V. parahaemolyticus* biofilms [7,78,79,80].

It is unlikely that quercetin will enter cells and directly interact with transcriptional regulators or intracellular objectives. According to our hypothesis, quercetin might interact with specific membrane proteins, which would then activate the bacterial signaling system and result in transcriptional changes that result in the downregulation of genes. Among other macromolecules, such as microbial adhesins and cell membrane proteins, quercetin, a polyhydroxy hydrolytic chemical, has the potential to create powerful complexes. The modifications to the membrane may lead the bacterial cells to adapt, modifying how their genes are expressed using bacterial signaling processes such as two-component systems.

## 5. Conclusions

We demonstrated quercetin’s effective antibacterial and perhaps anti-pathogenicity properties against *V. parahaemolyticus* on surfaces in contact with food. Additionally, quercetin considerably decreased the number of bacterial cells that were alive, broke up cell-to-cell connections and existing biofilms, and significantly decreased the expression of genes related to motility, virulence, and QS. In order to regulate the biofilm of *V. parahaemolyticus* in food-contact surfaces and reduce the risk of foodborne disease caused by this pathogen, quercetin may thus be developed as an alternative strategy.

## Figures and Tables

**Table 1:** Primer lists used in this study for RT-qPCR. F and R stand for forward and reverse primers.

Target Gene	Sequence of Primers (5′-3′)	Product Size (bp)	NCBI Accessions No.
*flaA*	F: CGGACTAAACCGTATCGCTGAAA R: GGCTGCCCATAGAAAGCATTACA	128	GQ433373.1
*flgL*	F: CGTCAGCGTCCACCACTT R: GCGGCTCTGACTTACTGCTA	141	CP066246.1
*luxS*	F: GGATTTTGTTCTGGCTTTCCACTT R: GGGATGTCGCACTGGTTTTTAC	119	CP066246.1
*aphA*	F: ACACCCAACCGTTCGTGATG R: GTTGAAGGCGTTGCGTAGTAAG	162	CP066246.1
*vp0952*	F: TATGATGGTGTTTGGTGC R: TGTTTTTCTGAGCGTTTC	276	CP064041.1
*vp0962*	F: GACCAAGACCCAGTGAGA R: GGTAAAGCCAGCAAAGTT	358	CP064041.1
*VopQ*	F: CCACAAGTTTGCTTCGGTTAG R: GGTTCTCCTCGGTAGCCTGA	174	AP026555.1
*Vpa0450*	F: TTGCTGAAGGCTCTGATG R: CTGCACTGGCTTATGGTC	275	AP026556.1
*16S rRNA*	F: TATCCTTGTTTGCCAGCGAG R: CTACGACGCACTTTTTGGGA	186	CP085308.1

## Data Availability

Data is contained within the article.
